# Functional beverage of *Garcinia mangostana* (mangosteen) enhances plasma antioxidant capacity in healthy adults

**DOI:** 10.1002/fsn3.187

**Published:** 2014-12-12

**Authors:** Zhuohong Xie, Marsha Sintara, Tony Chang, Boxin Ou

**Affiliations:** International Chemistry Testing258 Main Street, Suite 202, Milford, Massachusetts

**Keywords:** Aloe vera, bioavailability, green tea, LC/MS, mangosteen, ORAC

## Abstract

This study was to investigate the absorption and antioxidant effect of a mangosteen-based functional beverage in humans. The beverage contained mangosteen, aloe vera, green tea, and multivitamins. A randomized, double-blind, placebo-controlled clinical trial was conducted with generally healthy male and female subjects between 18 and 60 years of age. Ten men and 10 women participated in this study. Participants were randomly divided into two groups, treatment and placebo group. Participants received either a daily single dose (245 mL) of the beverage or a placebo. Blood samples were collected from each participant at time points 0, 1, 2, 4, and 6 h. The plasma samples were analyzed by LC/MS for *α*-mangostin and vitamins B_2_ and B_5._ Results indicated that the three analytes were bioavailable, with observed *C*_max_ at around 1 h. The antioxidant capacity measured with the oxygen radical absorbance capacity (ORAC) assay was increased with a maximum effect of 60% after 1 h, and the elevated antioxidant level lasted at least 6 h. This study demonstrated the bioavailability of *α*-mangostin and B vitamins from a xanthone-rich beverage and the mechanisms of the increase in plasma antioxidant may be direct effects from antioxidants, enhancement of endogenous antioxidant activity through activation of Nrf2 pathway, and synergism of the antioxidants.

## Introduction

There is overwhelming evidence of the importance of antioxidants to scavenge the reactive oxygen/nitrogen species (ROS/RNS) which are known to be involved in the pathogenesis of aging and many common diseases (Halliwell 1991, 1996; Ames et al. 1993). Based on the review conducted by the Institute of Medicine, a dietary antioxidant is defined as “a substance in foods that significantly decreases the adverse effects of reactive species, such as ROS and RNS, on normal physiological function in humans” (Monsen 2000). It is believed that consumption of tropical fruits can reduce the incidence of certain degenerative diseases, the protective effect is considered mainly to be due to the presence of various phyto-bioactives in fruits such as antioxidants (Lim et al. 2007; Alothman et al. 2009; Rufino et al. 2010). *Garcinia mangostana* L. (Clusiaceae), commonly known as mangosteen, is a slow-growing tropical evergreen tree mainly found in India, Myanmar, Sri Lanka, and Thailand. Mangosteen has dark purple to red-purple fruits. The edible fruit aril is white, soft, and juicy with a sweet taste. Recently, mangosteen extract has been used as a botanical dietary supplement in the United States, as mangosteen peel has been reported to contain a variety of bioactive compounds with potential applications as therapeutic agents or as functional food additives such as phenolic acids, tannins, xanthones, anthocyanins, and other bioactive compounds (Mahabusarakam et al. 1987). Nowadays, there is a worldwide pursuit of designing new functional beverages and healthy food products based on tropical fruits (Sabbe et al. 2009; Granato et al. 2010; Schauss et al. 2010). In this sense, a design of new beverages combining mangosteen with green tea, aloe vera, and antioxidant vitamins was favorable on the basis of the antioxidant synergistic effects among these bioactives. The aim of this work was to determine the bioavailability of *α*-mangostin and vitamins B_2_ and B_5_ found in a mangosteen-based functional beverage and their antioxidant capacity on plasma antioxidant status in the human body.

## Materials

*α*-Mangostin was purchased from Chromadex (Irvine, CA), Vitamin B_2_ (riboflavin), Vitamin B_5_ (pantothenic acid), Trolox (6-hydroxy-2,5,7,8-tetramethyl-chroman-2-carboxylic acid), and Fluorescein sodium were obtained from Sigma-Aldrich (St. Louis, MO). 2,2′-Azobis (2-amidinopropane) dihydrochloride was purchased from Wako Chemicals USA (Richmond, VA). Verve® and placebo (fructose liquid) were provided by Vemma Nutrition Co. (Tempe, AZ). Verve® is a multivitamin/antioxidant liquid nutrition beverage containing a full spectrum of vitamins, green tea, aloe vera and mangosteen, and a caffeinated energy blend.

### Subjects and study protocol

A randomized, double-blind, placebo-controlled clinical trial was conducted with generally healthy male and female subjects between 18 and 60 years of age. All subjects were screened by evaluation of a medical history and assessment of diet history and supplement history using a self-developed semiquantitative questionnaire. Written informed consent was obtained from each volunteer participating in this study. All procedures of the protocol were approved by the Institutional Review Board of HummingbirdIRB (Cambridge, MA). Ten men and 10 women participated in this study. Participants were randomly divided into two groups, treatment and placebo group, with the same number of male and female participants in each group, and the trial duration was 6 h. After the baseline tests were completed in the morning, participants received either a daily single dose (245 mL) of the Verve formula or a placebo (a liquid that looks and tastes like Verve, but with no active ingredients). The use of fructose was to mimic the taste of Verve with minimal interference with absorption and availability of vitamins and phytonutrients. Blood samples were collected from each participant after consumption of the Verve formula or the placebo at time points 1, 2, 4, and 6 h. Plasma was obtained by centrifugation and stored at −80°C until further analysis.

### LC-MS/MS analysis of plasma samples

**Figure 1:**
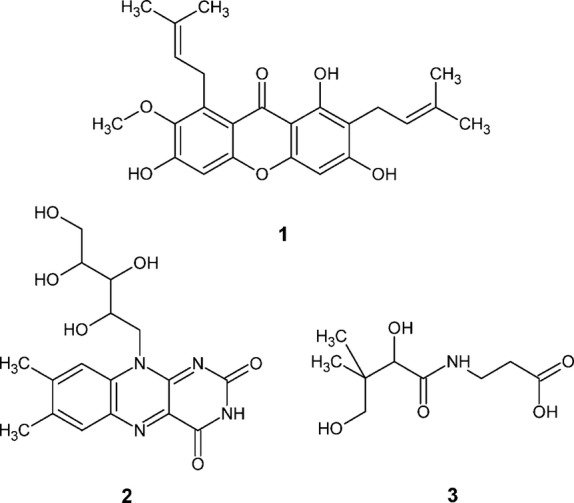
Structures of test analytes: 1, *α*-mangostin; 2, vitamin B2; 3, vitamin B5.

LC-MS/MS analyses were performed using a system consisting of an Agilent 1100 system comprising a binary pump with a vacuum degasser, a thermostatted column compartment, a C18 column, an autosampler, and a diode array detector (Agilent Technologies, Inc., Santa Clara, CA), coupled with an API-3000 mass spectrometer with a turbo ion spray source (Applied Biosystem, Foster City, CA). Sample preparations and conditions for LC-MS analyses were as given below. The chemical structures of test analytes are illustrated in Figure1.

### *α*-Mangostin

**Figure 2:**
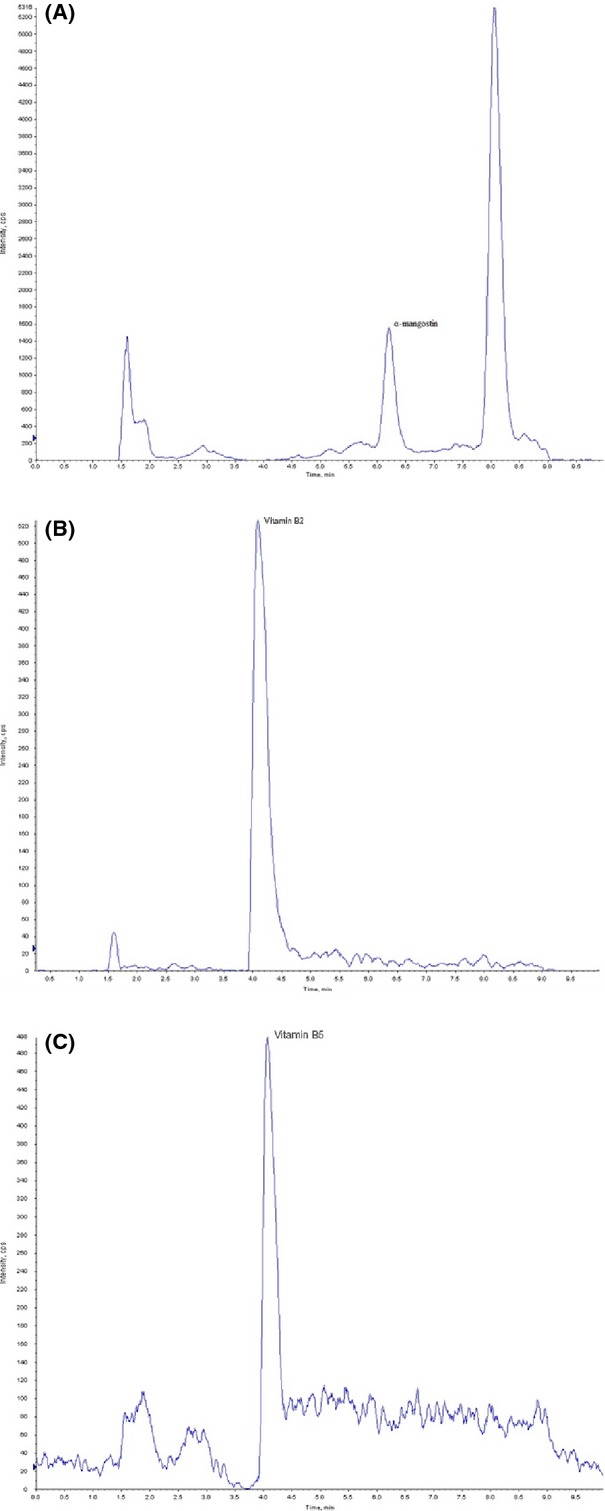
Representative chromatograms of MRM scans for (A) a-Mangostin, (B) vitamin B2, and (C) vitamin B5. MRM, multiple reaction monitoring mode.

One hundred microliters of reconstituted human plasma was extracted using 300 *μ*L of methanol. The supernatant was collected after centrifugation at 14,000 rpm for 10 min (4°C). Ten microliters was injected onto the LC-MS/MS for mangostin analysis. Chromatography was carried out on a 2.1 × 50 mm, 3 *μ*m, MacMod HyroBond PS-C18 column (MAC-MOD Analytical, Inc., Chadds Ford, PA), and the solvent system consisted of a gradient system with water (0.4% formic acid, v/v) (A) and acetonitrile (B). Gradient elution was performed at 0.6 mL/min with the following conditions: 1 min hold at 100% A, 0.5 min linear gradient from 100% to 60% A, 4.5 min linear gradient from 60% to 2% A, 1.1 min hold at 2% A, 0.1 min linear gradient from 2% to 100% A, and 2.9 min hold at 100% A. The mass detector was equipped with a turbo ion spray (electrospray ionization, ESI) source and operated in multiple reaction monitoring mode (MRM) under negative ion mode. The heated capillary and voltage were maintained at 550°C and −4500 V, respectively. The optimized instrument setting is listed in Table1. Representative chromatograms of MRM scans are shown in Figure2. The lower limit of quantification (LLOQ) for *α*-mangostin was 1.0 ng/mL.

**Table 1:** Mass spectrometry parameter for *α*-mangostin, vitamin B2, and vitamin B5

		Transition (m/z)				
Analyte	MW	Parent ion	Product ion	DP (V)	CE (V)	CXP (V)
*α*-Mangostin	410.46	411.3	355.3	61	23	4
Vitamin B2	376.36	377	243.2	96	33	14
Vitamin B5	219.23	220.3	90.1	41	21	6

DP: De-clustering potential;

CE: Collision Energy;

CXP:Collision Cell Exit Potential;

MW: Molecular Weight.

### Vitamins B_2_ and B_5_

Two hundred microliters of reconstituted human plasma was mixed with 280 *μ*L of methanol and 20 *μ*L of trifluoroacetic acid. The supernatant was collected after vortexing and centrifugation at 14,000 rpm for 10 min (4°C). Five microliters was injected onto the LC-MS/MS for vitamin B analysis. Chromatography was carried out on a 50 × 4.6 mm, 3 *μ*m, Ascentis RP-AMIDE column (Sigma-Aldrich), and the solvent system consisted of a gradient system with water (0.4% formic acid, v/v) (A) and acetonitrile (B). Gradient elution was performed at 0.6 mL/min with the following conditions: 0.9 min hold at 100% A, 0.1 min linear gradient from 100% to 90% A, 3.0 min linear gradient from 90% to 40% A, 1 min linear gradient from 40% to 2% A, 2.0 min hold at 2% A, 0.1 min linear gradient from 2% to 100% A, and 3.0 min hold at 100% A. The mass detector was equipped with a turbo ion spray (ESI) source and operated in MRM under positive ion mode. The heated capillary and voltage were maintained at 550°C and 5500 V, respectively. The optimized instrument settings for vitamins B_2_ and B_5_ analysis are listed in Table1. Representative chromatograms of MRM scans are shown in Figure2. The LLOQs for vitamins B_2_ and B_5_ were 2 and 13 ng/mL, respectively.

### Antioxidant capacity (ORAC Assay)

Plasma antioxidant capacity was determined by the ORAC assay as previously described using a Synergy H4 microplate florescence reader (Bio-Tek Instruments, Inc., Winooski, VT) with Gen 5 V 2 software (excitation wavelength 485 ± 20 nm, emission wavelength 530 ± 25 nm) (Ou et al. 2001; Prior et al. 2003). Plasma samples were thawed, vortexed, and centrifuged at 16,000*g* at 4°C for 3 min, 240 *μ*L of supernatant was deproteinized using 720 *μ*L of methanol. The mixture was vortexed for 30 sec, and then centrifuged at 16,000 *g* for 5 min at 4°C. Peroxyl radicals were generated by the spontaneous decomposition of AAPH at 37°C. Fluorescein was used as a fluorescent probe, with loss of fluorescence indicating fluorescein damage from its reaction with peroxyl radicals. The protective effects of the plasma tested were determined by comparing fluorescence time/intensity area under the curve (AUC) of the sample with a control.

## Results and Discussion

### Bioavailability

**Figure 3:**
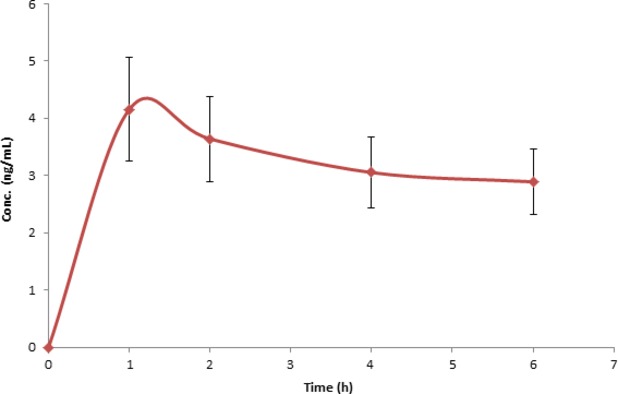
Concentrations of *α*-mangostin in human plasma vs. time curve after a single oral administration of Verve® (245 mL). Values were expressed as means ± SEM.

A variety of health-promoting attributes have been associated with the mangosteen, which include antiinflammatory, antibacterial activity, cardioprotective, and antioxidant activity. A class of compounds known as xanthones have been isolated from mangosteen which are thought to be responsible for mangosteen's bioactivity. Of all the xanthones, *α*-mangostin (1,3,6-Trihydroxy-7-methoxy-2,8-bis(3-methyl-2-butenyl)-9H-xanthen-9-one) has been identified as the most abundant xanthone and as a result has received the most attention for its health-promoting properties. The plasma level of *α*-mangostin was analyzed in this study. As demonstrated in Figure3, an increase of *α*-mangostin was observed during the course in the treatment group, whereas no *α*-mangostin was found in the placebo group within the detection range. The *α*-mangostin plasma concentration reached its maximum (*C*_max_), 4.16 ± 2.85 ng/mL at *t*_max_ of 1 h after Verva formula consumption, the elevated level of *α*-mangostin was sustained through the rest of course of the trial.

**Figure 4:**
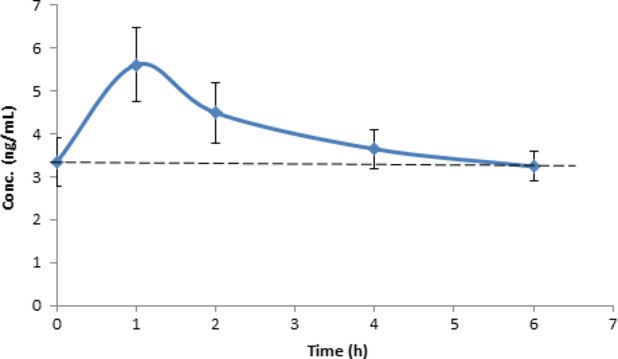
Concentrations of vitamin B_2_ in human plasma vs. time curve after a single oral administration of Verve® (245 mL). Values were expressed as means ± SEM. Dashed line represents baseline level.

**Figure 5:**
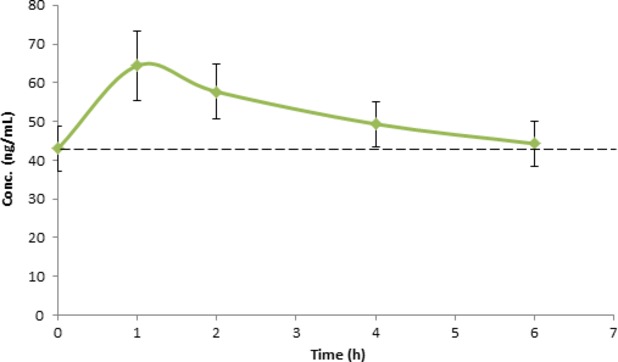
Concentrations of vitamin B_5_ in human plasma vs. time curve after a single oral administration of Verve® (245 mL). Values were expressed as means ± SEM. Dashed line represents baseline level.

Verva formula is enriched with vitamins B_2_ and B_5_. B vitamins are essential nutrients and important for cell metabolism, therefore, they are vital as part of one's health promotion. In this study, we also evaluated the bioavailability of B vitamins. Figures4 and 5 illustrate the mean plasma concentration–time curves of tested analytes. As shown, both vitamin B_2_ and B_5_ were found bioavailable, and after 1 h their concentrations in plasma reached the highest level at 5.61 ± 2.59 ng/mL and 64.41 ± 26.89 ng/mL, respectively, this trend was aligned with the absorption of *α*-mangostin.

### Antioxidant capacity

**Figure 6:**
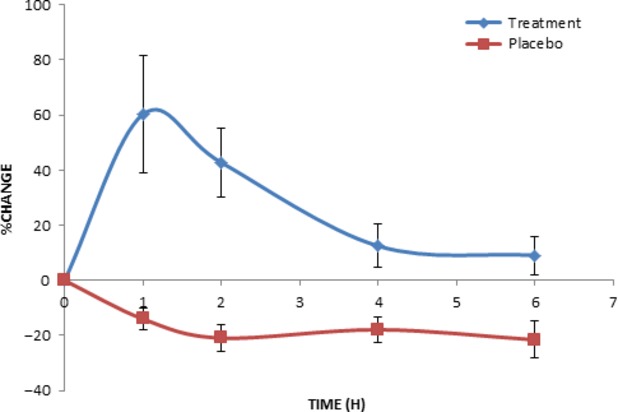
Increase of human plasma antioxidant capacity after a single oral administration of Verve® (245 mL). Values were expressed as means ± SEM.

The plasma antioxidant capacity (ORAC) in the Verva formula group increased as much as 60% at 1 h following consumption of the drink (Fig.6). The ORAC values then declined gradually and became stable between 4 and 6 h with 10% increase when compared to the zero hour. While in the placebo group, the antioxidant activity in plasma was found slightly decreased over the trial period.

### Mechanism of plasma ORAC increase

The increases in plasma B vitamins and *α*-mangostin following Verve consumption indicate that at least a small part of the increase in ORAC may be accounted for by absorbed B vitamins and *α*-mangostin. Additionally, Verva also contains vitamin E, vitamin C, green tea extract, and aloe vera. Vitamins C and E are well-known antioxidants, and green tea catechins belong to flavonoid family and are known to have fairly strong antioxidant activity with ORAC values of 8100−22,000 *μ*mol of Trolox equiv/g (Ou et al. 2002). Polysaccharides from aloe vera scavenge hydroxyl radicals, the most harmful reactive oxygen species found in the body (Hu et al. 2003; Kardošová and Machová 2006; Miranda et al. 2009). Hence, it is reasonable to propose that vitamins C and E, *α*-mangostin, green tea catechins, and aloe vera polysaccharides might account for the increase in plasma ORAC.

On the basis of the nanogram levels of absorbed B vitamins and *α*-mangostin, the direct effects from the antioxidants on the increase in ORAC are therefore limited. The human body's endogenous antioxidant network consists of several enzymatic antioxidants, such as glutathione, thioredoxin, and NADPH, etc (Halliwell 1996). Recently, antioxidants present in fruit and vegetables are found to act together to produce an additive increase in electrophilic signaling that results in the induction of endogenous antioxidant responses to oxidative stress (Su et al. 2013). The upregulation of endogenous antioxidant defense provides the more profound cellular protection than that of antioxidant supplementation. This is because some phytochemicals can activate the transcription factor nuclear factor (erythroid-derived 2)-like 2 (Nrf2), a “master regulator” of antioxidant defense (Jung and Kwak 2010; Zhang et al. 2010; Lee et al. 2013). Shen et al. (2014) reported that *α*-mangostin-mediated antioxidant response via Nrf2 is a mechanism preventing adipogenesis and inflammation in adipocytes. Molecular mechanisms underlying antioxidant activity exerted by green tea and its components have been extensively investigated as well. (−)-Epigallocatechin-3-gallate (EGCG), a major green tea catechins, has been shown to induce expression of endogenous enzymatic antioxidants such as glutathione *S*-transferase, glutathione peroxidase, glutamate cysteine ligase, hemeoxygenase-1, etc. (Na and Surh 2008). Vitamin E is a well-known antioxidant compound that inhibits lipid peroxidation and other free radical-mediated reactions in biological systems (Niki 2014). In an in vivo study Cardozo et al. (2013) observed that Nrf2 was significantly upregulated following consumption of a vitamin E- rich diet. Furthermore Yang et al. (2014) found that wolfberry polysaccharide showed the antioxidative and anti-inflammatory effects on experimental models of insulin resistance in vivo, one of the mechanisms was determined to be induction of Nrf2 Pathway. Taken together, these studies suggest that phytochemicals have the ability to activate the Nrf2 system, which in turn, increases the activity of antioxidant enzymes and inhibits oxidative stress. Although the molecular mechanism of the observed antioxidant effect was not studied, we would expect that the increase in enzymatic antioxidant activity through activation of Nrf2 might have been taken in place.

The additive and synergetic effects of phytochemicals in antioxidant activity have been well studied (Liu 2003; Seeram et al. 2004; Chen et al. 2005; Milde et al. 2007; Mikstacka et al. 2010; Wang et al. 2011). Liu et al. (2000) found that the antioxidant activity of vitamin E could be enhanced by green tea extract, as catechins in green tea can effectively reduce *α*-tocopheroxyl radicals to *α*-tocopherol, prolonging the antioxidant-free radical chain breaking reaction. In addition, the combination of vitamin C and vitamin E results in more effective in inhibiting oxidation in vivo, this is because coexisting vitamin C reduces *α*-tocopheroxyl radicals rapidly in membranes and LDL to regenerate *α*-tocopherol and possibly inhibits *α*-tocopheroxyl radicals initiated propagation (Sato et al. 1990). As demonstrated above, the direct effects from individual antioxidants contributed to the increase in plasma ORAC; however, the significant plasma ORAC after consumption of Verve in a single dose cannot be interpreted by single antioxidant. It is logical to propose that the observed ORAC increase may be partially due to the synergism of the combination of mangosteen, green tea, antioxidant vitamins, and aloe vera polysaccharides. It is widely believed that the health benefit of a diet rich in fruits and vegetables is attributed to the complex mixture of plant antioxidants present in these and other whole foods, no single antioxidant can replace the combination of phytochemicals in achieving the optimal health benefits (Liu 2003). In plant extracts and in various foods, antioxidants are present as mixtures and therefore there is currently a great interest in synergistic interaction between antioxidants and between mechanisms of such interaction (Almeida et al. 2011). It appears that Verve is formulated based on the balanced antioxidants combining water-soluble antioxidants (vitamin C, catechins, and polysaccharides) and fat soluble antioxidants (vitamin E and *α*-mangostin). Therefore, the synergy between water soluble and fat soluble antioxidants may provide at least one of the explanations to Verve's antioxidant health benefit.

In summary, we determined the physiological availability of *α*-mangostin and B vitamins present in a xanthone-rich functional beverage, and their effects on the degree of antioxidant potency in the human body. Noticeable bioavailability was seen in *α*-mangostin and vitamins B_2_ and B_5_ along with the increase in antioxidant capacity. In the experiment group within 1 h after consumption of Verva, significant increases of *α*-mangostin, vitamins B_2_ and B_5_, and ORAC were observed in these subjects. In the placebo group, no significant change was observed in vitamins B_2_ and B_5_, and no *α*-mangostin was detected. Based on the observation of the result and literature, we proposed that the increase in plasma ORAC may be due to three mechanisms: First, bioavailable antioxidants directly scavenged free radicals. Second, phytochemicals upregulated Nrf2 signaling pathway, resulting in increase in endogenous antioxidant activity. Third, the synergistic and additive effects among the antioxidants may account for plasma ORAC. Our results suggest that Verve is an excellent source to obtain balanced natural antioxidants to maintain well-being and possibly against chronic diseases caused by the aging process.

## Conflict of Interest

None declared.
